# Uniaxial Tensile Behavior of Carbon Textile Reinforced Mortar

**DOI:** 10.3390/ma12030374

**Published:** 2019-01-25

**Authors:** Fen Zhou, Huanhui Liu, Yunxing Du, Lingling Liu, Deju Zhu, Wei Pan

**Affiliations:** 1 Key Laboratory for Green & Advanced Civil Engineering Materials and Application Technology of Hunan Province, College of Civil Engineering, Hunan University, Changsha 410082, China; duyunxing@hnu.edu.cn (Y.D.); dzhu@hnu.edu.cn (D.Z.); 2 Key Laboratory for Damage Diagnosis of Engineering Structures of Hunan Province, Hunan University, Changsha 410082, China; 3 College of Civil Engineering, Hunan University, Changsha 410082, China; liuhuanhui@hnu.edu.cn (H.L.); liulingling@hnu.edu.cn (L.L.); 4 Department of Civil Engineering, The University of Hong Kong, Pokfulam, Hong Kong, China; wpan@hku.hk

**Keywords:** carbon textile reinforced mortar, uniaxial tensile tests, debonding failure, steel fibers, prestress, multi-cracking pattern

## Abstract

This paper investigates the effects of the reinforcement ratio, volume fraction of steel fibers, and prestressing on the uniaxial tensile behavior of carbon textile reinforced mortar (CTRM) through uniaxial tensile tests. The results show that the tensile strength of CTRM specimens increases with the reinforcement ratio, however the textile–matrix bond strength becomes weaker and debonding can occur. Short steel fibers are able to improve the mechanical properties of the entire CTRM composite and provide additional “shear resistant ability” to enhance the textile–matrix bond strength, resulting in finer cracks with smaller spacing and width. Investigations into the fracture surfaces using an optical microscope clarify these inferences. Increases in first-crack stress and tensile strength are also observed in prestressed TRM specimens. In this study, the combination of 1% steel fibers and prestressing at 15% of the ultimate tensile strength of two-layer textiles is found to be the optimum configuration, producing the highest first-crack stress and tensile strength and the most reasonable multi-cracking pattern.

## 1. Introduction

Textile reinforced mortar (TRM), also called textile reinforced concrete (TRC) in some cases with slightly coarse aggregates in matrix, refers to an emerging type of cement-based composite material characterized by reinforcing high-performance, fine-grained concrete with high-ductility, alkali-resistant textiles [1,2,3]. It was born as a result of reinforcing and/or rehabilitating aging masonry and reinforced concrete in conventional structures. The combination of fine-grained concrete and high-strength textiles produces holistic composites that may enable the fabrication of sophisticated and lightweight concrete structures, stay-in-place formwork elements, and prefabricated sandwich panels with extraordinary mechanical performance, very high durability, and enhanced potential for free-form designs in comparison to steel-bar reinforced concrete [4,5,6]. Moreover, compared with engineered cementitious composites which have a relatively low fiber utilization rate because the short fibers are randomly distributed in the matrix [7,8,9], TRM has high-performance fibers that are continuously embedded in the mortar matrix along the anticipated principal stress direction which results in a higher fiber utilization rate with the same reinforcement ratio [10]. Therefore, TRM has gradually become an attractive replacement composite material for engineering applications [11]. With recent developments in civil engineering, TRM can also be used in the supporting and connecting components of new structures [12,13,14,15,16].

As TRM is increasingly applied in the construction industry, an in-depth understanding of the fundamental mechanism developed via experimental investigation becomes essential for analysis, modeling, and design. The following paragraphs describe some recent studies on the mechanical behavior of TRM.

A number of studies have shown that the textile reinforcement ratio significantly affects the tensile behavior of TRM. Contamine et al. [17] studied the direct tensile behavior of TRC composites that were produced through the laminating technique. The results showed that composites with high reinforcement ratios were insensitive to defects and, thus, provide reliable test results. Larrinaga et al. [18] observed that specimens reinforced with one layer of basalt textile broke smoothly and an increase in the reinforcement ratio turned the failure mode into a brittle rupture with a sudden load drop during tensile tests, indicating that there exists a critical threshold for positive effects: once a certain ratio has been reached, the potentiation may become weak and inadequate fracture modes may occur. A typical three-stage evolution theory depicting the relationship between the stress and strain of TRC has been unanimously approved. Initially developed in the 2000s at the Technische Universität Dresden [19,20], this theory indicates that TRC materials exhibit distinct strain-hardening behavior. Several researches [21,22] have further refined the tensile behavior of TRC using the classic three-stage stress–strain curve. 

With further research, it is gradually becoming clear that the load-bearing capacity of TRM is strongly related to the synergistic effect of the components, which is intensively affected by the bond property between the textile and the matrix [23]. A promising approach for improving the bond is to impregnate textiles with epoxy resin before producing the TRC composite, as reported by Dvorkin et al. [24]. Colombo et al. [25] then investigated the influences of the reinforcement ratio, textile geometry, curing condition, and specimen size on the mechanical properties of AR-glass TRC. The results revealed that the bond strength between textiles and matrix tends to increase with the increasing number of textile layers, weft spacing, and shrinkage caused by different curing conditions; consequently, both the first-crack stress and tensile strength increase. Although the increasing specimen size enhances ductility, no significant changes in tensile strength have been observed. 

To further improve the mechanical performance of TRM composites, process modifications and some exterior additions have been developed. In terms of process modifications, pre-tensioning turns out to be an effective approach. Reinhardt et al. [26] showed that the application of prestressing on textiles improved the cracking, tensile strength, and stiffness of cracked sections, with more notable effects occurring in impregnated carbon TRC specimens. As a result, the prestressing process can significantly extend the serviceability of TRC composite materials. With respect to the exterior additions, Barhum and Mechtcherine [27] addressed the influence of short dispersed fibers made of AR glass on the fracture behavior of TRC by uniaxial tests. It is reported that TRC specimens that are reinforced with short dispersed fibers enhance the first-crack stress (by a factor of 2) and form more and finer cracks. Du et al. [28] explored the flexural behavior of basalt textile reinforced concrete (BTRC) with a combination of prestress and chopped steel fibers and found that chopped steel fibers increase the crack number of BTRC specimens; this effect was more obvious at higher prestress levels. 

Textiles made from popular fibers, including synthetic groups such as AR (alkali resistant) glass, basalt, carbon, or aramid [29,30] and natural groups such as sisal, hemp, and flax [31,32], have received the most attention from researchers. Carbon textile has been found to provide better supported load capacity and higher strength and Young’s modulus when used as traction reinforcement [33,34].

Despite significant efforts to investigate the mechanical properties of TRM composite materials, limited information is available regarding the effects of adding steel fibers with different volume fractions and applying different levels of prestressing force to carbon textile reinforced mortar (CTRM). In particular, no relevant experimental data are available for the uniaxial tensile behavior of prestressed CTRM composites with the addition of steel fibers, which are known to offer superior tensile strength and have a high elastic modulus [35]. The present research aims to investigate the influence of the textile reinforcement ratio, volume fraction of short steel fibers, and prestressing force on the uniaxial tensile behavior of TRM. In the following sections of this paper, the main materials considered in this study, including carbon textiles, short steel fibers, and fine-grained mortar, are described, and the pre-tensioning of textiles is explained in detail. The experimental profiles and corresponding test results are presented, and the differences in tensile properties (including first-crack stress, tensile strength, crack numbers, and crack spacing) according to the three design variables are discussed. An optical microscope is used to illustrate the distinctions among the failure modes of the test specimens.

## 2. Materials and Methods

### 2.1. Materials

#### 2.1.1. Carbon Textile

**Figure 1:**
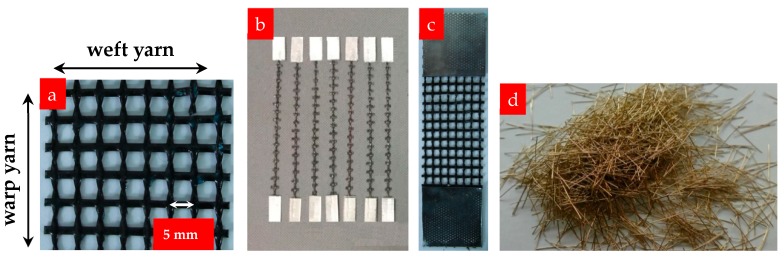
(**a**) Carbon textile impregnated with resin epoxy, (**b**) single yarn samples, (**c**) strip sample, and (**d**) steel fibers.

The carbon textile that was used in this study is manufactured in two orthogonal directions at a nominal spacing of 5 mm. Multifilament yarns consisting of 6000 monofilaments are used to produce the textile. The outer filaments (sleeve filaments) are in direct contact with the matrix and, therefore, have a greater effect on the bond properties than the inner filaments (core filaments). The inner filaments have no direct contact with the matrix because of the low penetration of the matrix. The force on the sleeve filaments is transformed to the core filaments via friction; thus, the untreated textiles exhibit little cooperative bearing ability between the sleeve and core filaments. A large number of experimental studies have shown that epoxy resin can fully penetrate all filaments within the yarns, thereby providing an intact unit reinforcing system [36,37]. Hence, impregnated carbon textiles are used in this study, and the warp yarns (along the length of the textile) are considered to be the reinforcing yarns, as shown in Figure 1a.

Details about the physical and mechanical properties of the impregnated carbon yarns (reinforcing direction) are presented in Table 1. The tensile strength, Young’s modulus, and elongation of the carbon yarns were determined through direct tensile tests using 100-mm gauge length samples according to the Chinese specification GB/T 3362-2017 (Figure 1b). The cross-sectional area of a single yarn is 0.218 mm^2^, calculated as the ratio of tex (the linear density of this material) to its bulk density. Ten 40-mm wide strips of 100-mm gauge length samples were cut from the textiles (Figure 1c) to determine their tensile properties. The average tensile bearing capacity and tensile strength of the 10 test strips were 4 kN and 2293.6 MPa, respectively, as determined via tensile tests.

#### 2.1.2. Short Steel Fibers

In view of the limitations of the mesh size of carbon textiles and the mold height (20 mm), copper-coated steel fibers of length 12–15 mm were used, as shown in Figure 1d. The mechanical properties of the steel fibers are listed in Table 2. The density was obtained by dividing the mass by the volume, and the volume was measured using the drainage method.

#### 2.1.3. High-Performed Fine-Grained Mortar

The matrix of TRM specimens ought to have super fluidity and self-compactness, so the maximum particle size of the aggregates should be less than 2 mm. Given that textiles in some specimens were pre-tensioned, the matrix should also possess high early strength in order to reduce the loss of prestress. Therefore, the mix proportion of high-performance fine-grained mortar designed by Du et al. [35] was used (see Table 3). The fly ash improves the fluidity and the silica fume and slag ash are designed to improve the early strength. Samples with dimensions of 40 mm × 40 mm × 160 mm were produced to study the mechanical properties of the matrix, especially the early mechanical properties. The flexural strength of the matrix was measured through three-point bending tests, and the values after 7 and 28 days were 11.5 MPa and 12.3 MPa, respectively. The compressive strength of the matrix was determined on the two broken parts that were obtained from the three-point bending tests, and the values after 7 and 28 days were 62.5 MPa and 76.7 MPa, respectively, indicating that the mortar matrix prepared according to the designed mix proportion satisfied the high early strength requirement.

### 2.2. Experimental Program

#### 2.2.1. Testing Series

In this study, the tensile performance of carbon TRM was examined with respect to the reinforcement ratio of the textile, the addition of steel fibers at different volume fractions, and the prestressing force applied to the carbon textiles. The specific test schemes included two primary specimen series in which TRM composites that were reinforced with one or two textile layers were produced; control specimens with no textile layers were also produced.

The reinforcement ratios of the specimens that were produced with one and two layers of textile are 0.4% and 0.8%, respectively. The reinforcement ratio can be expressed as follows:

(1) $$\rho_{f}=\frac{A_{f}}{A_{c}}$$

where $A_{f}$ and $A_{c}$ are the cross-sectional areas of the carbon yarns and TRM specimens, respectively. 

The effects of steel fibers with different volume fractions (0.5%, 1%, and 2%) were studied in each series, and the effects of prestress on the mechanical behavior of the TRM composites were investigated by applying different prestressing forces to the one-layer (10% and 20% of the ultimate tensile strength of one-layer textile) and two-layer (15% of the ultimate tensile strength of two-layer textiles) specimens. Moreover, prestressed thin plates with 1% steel fibers added were researched to determine their effects on the mechanical behavior. The test specimens were named as follows: for example, P15C2S1 represents specimens with a prestress of 15% of the ultimate tensile strength of two-layer textiles (P15), a two-layer carbon textile arrangement (C2), and the addition of 1% (by volume) steel fibers (S1).

#### 2.2.2. Tensioning System

**Figure 2:**
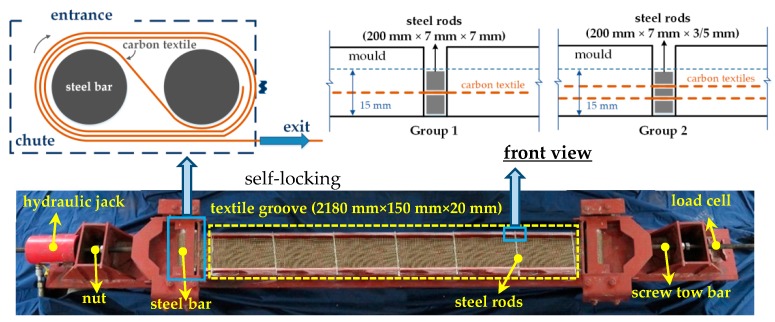
Illustration of the tensioning system.

Figure 2 shows the device that was used to apply pre-tensioning to the carbon textiles. Considering that the pre-tensioning will decrease slightly over time, the pre-tensioning of the carbon textiles should be completed before casting the mortar matrix. The device is employed as follows:

1. Firstly, the two free ends of the carbon textiles, which are not impregnated with the epoxy resin within the range of around 80 mm, are anchored at either end of the device using the self-locking principle (see Figure 2). During the process, the roller beneath the chute slides to adjust the position of the chute. Note that warp fibers in the upper and lower layers (when there are two textile layers) must be strictly aligned and parallel to the edge of the textile groove.
2. Stretch the carbon textiles by tightening the nut at one end. Note that the loading process should be uniform and slow so that the fiber bundles are evenly and cooperatively stressed. The pre-tensioning force is measured by the load cell at the other end, output to the data acquisition system, and is finally displayed on the digital terminal.
3. When the target pre-tension is reached, the loading is paused. After 5–10 minutes, the loss of pre-tension is measured and recorded. The pre-tension force is then re-applied to reach the target value. This operation calls additional tensioning, which should be repeated afterwards. To maintain a stable pre-tension level, the textile should stay stretched for 24 h, during which time additional tensioning should be implemented every 8 h; in other words, the additional tensioning is repeated three times in total.
4. Finally, the mortar matrix is cast on the textile.

#### 2.2.3. Preparation of the Composite Specimens

The manufacturing process started with all textiles tautly positioned on the groove (2180 mm × 150 mm × 20 mm) of the tensioning system (Figure 2), i.e., one layer of textile was positioned at the middle height of the specimen or two layers of textiles were placed uniformly along the height of the specimen. Two groups of overlapping steel rods (thicknesses of 3, 5, 7 mm) were used to determine the location of the textile layers and the thickness of the specimens (Figure 2). Note that the global thickness of all specimens was set to 15 mm.

**Figure 3:**
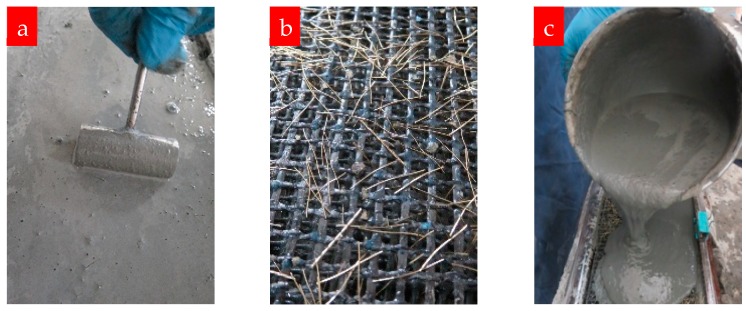
(**a**) Surface treatment of TRM composite, (**b**) steel fibers inserted into the grids of the textile, (**c**) flowable fresh mortar matrix with steel fibers.

Firstly, the carbon textiles were fixed in the groove and slightly stretched. As for prestressed specimens, the textiles must be tensioned as described in Section 2.2.2. The fresh mortar matrix was then poured into the groove and a flat vibrator was used to eliminate pores; the top surface was later smoothed by a roller (Figure 3a). After the surface of the mortar had hardened, the plate was covered with wet towels and cured at room temperature. The prestressed plates were cured for four days before being released and demolded, whereas the other specimens were only cured for one day before demolding. The prestressing force was released by cutting off the textiles in a tensioned state, and then the plates were removed from the molds and stored in a climate-controlled room at 20 °C and 90% relative humidity until the age of 28 days. The TRM plates were then cut into specimens with dimensions of 240 mm × 40 mm (length × width) using a water-cooled cutting machine. For each test condition, there were at least six valid specimens. As soon as the specimens had dried, a thin layer of white paint was applied to the surface to aid the observation of the crack pattern.

Figure 3b,c show the distributions of steel fibers. To facilitate more uniform distributions of steel fibers in the matrix and effective stress transmission between the textile and matrix, some of the steel fibers were inserted vertically or obliquely into the textile grids, and the remainder were thoroughly mixed up with the mortar matrix and then poured into the mold.

#### 2.2.4. Uniaxial Tensile Test Setup

**Figure 4:**
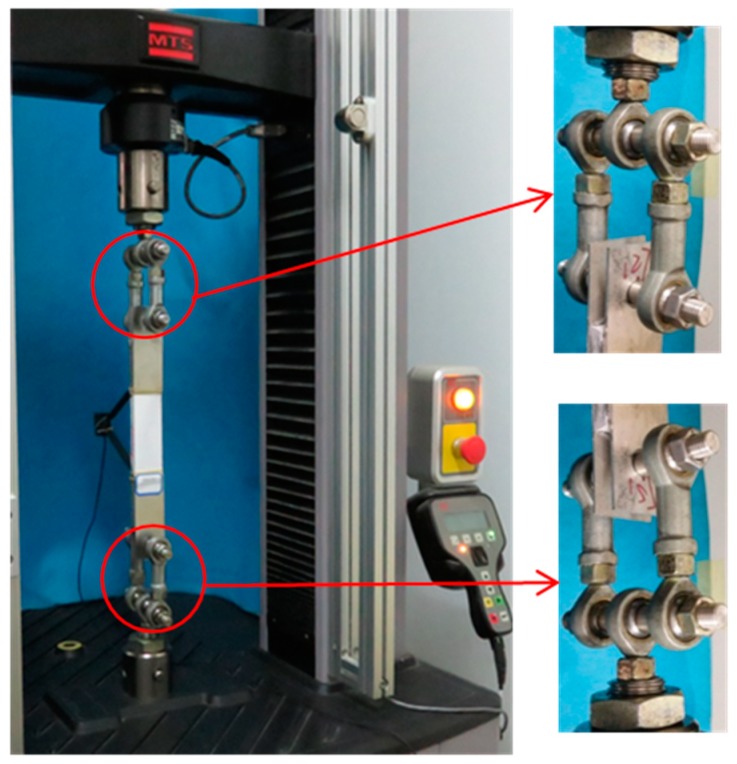
Uniaxial tensile test setup.

**Figure 5:**
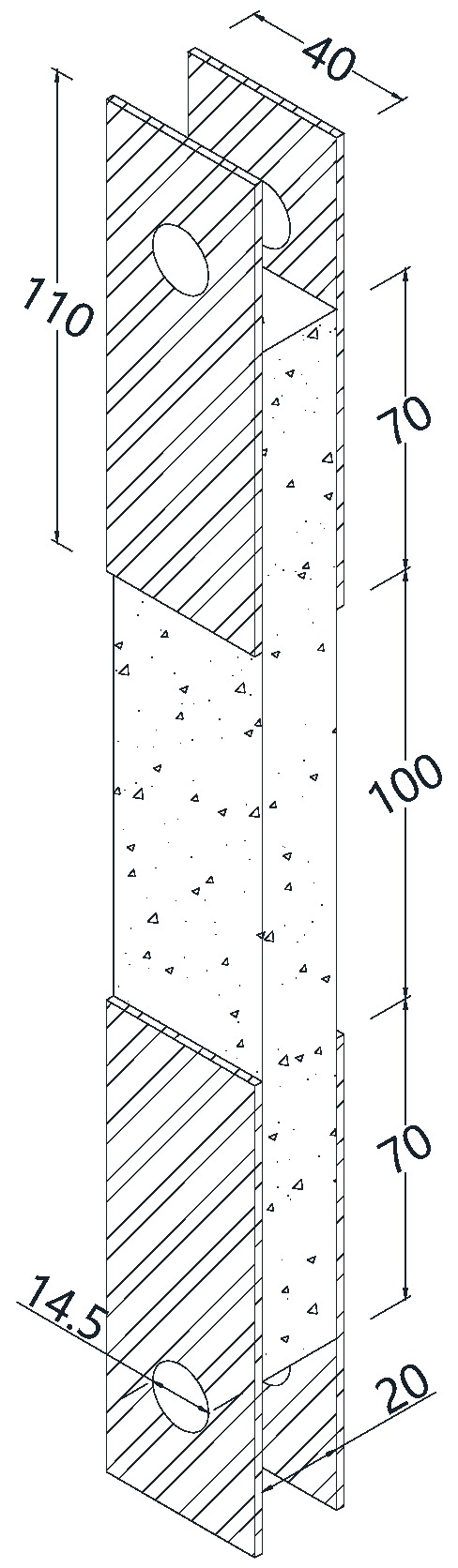
Dimensions of the TRM specimens (units: mm).

Uniaxial tensile tests were performed using an MTS (MTS System Corporation, Shenzhen, China) load frame (C43.304) with a load capacity of 30 kN and a maximum sampling rate of 1000 Hz (Figure 4). In this study, the tests were carried out by displacement control (0.5 mm/min) with a sampling rate of 20 Hz. The deformations were measured using an extensometer with a gauge length of 100 mm positioned in the central area of the specimens. Both the loads and deformations were recorded simultaneously through a computer that was connected to the testing machine. The stress was calculated by dividing the load by the cross-section of the specimen. A simple and cost-effective device was used to avoid the negative effects of possible eccentricity and misalignment, as shown in Figure 4. Five-ball joints were installed at both the top and bottom of the machine grips as universal joints, and 2-mm-thick aluminum plates with 14.5-mm diameter pinholes were attached to both ends of the specimens (Figure 5); the specimens were connected to the universal joints with a 14.5 mm diameter pin (Figure 5). Using this method, the applied axial load could be transformed from the machine grips to the specimens without causing bending effects [17,38]. Notably, the pin and pinhole must fit perfectly to prevent any rotation of the specimen at the beginning of the test. The specimens had a preload of 15 N applied. To observe the crack development, a digital camera was used to record the cracks during the loading process at intervals of 15 s.

## 3. Results and Discussion

**Figure 6:**
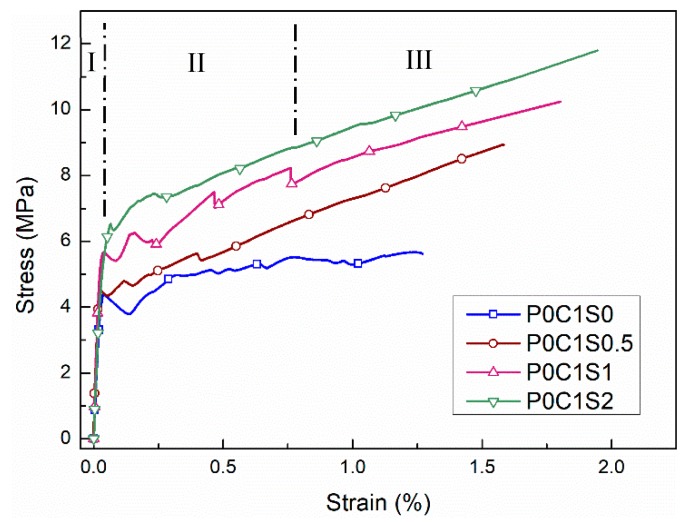
Typical tensile stress—strain curves of TRM specimens.

Representative curves from each testing protocol, showing the relationship between the stress and strain of the test specimens, are displayed in Figure 6. They are in good agreement with previous three-stage TRM stress–strain curves identified under tensile testing [18,39]. In stage I, the stiffness and volume proportion of both the matrix and textiles determine the slope of the curve, and the matrix plays a decisive role. In this stage, TRM exhibits nearly linear-elastic behavior until the point at which the stress reaches the tensile strength of the matrix, leading to the formation of the first crack. Stage II corresponds to a multi-cracking stage: the length and slope of this part of the stress–strain curve depend on the quality of the textile–matrix bond properties. Stage III is regarded as a strain-hardening stage and is characterized by high tensile strength and high strain capacity. In this stage, the existing cracks continuously widen and few further cracks appear. Furthermore, TRM exhibits linear behavior in this region, with the textiles carrying the whole load until the composite fails. In several tests where the transition from the second to third stages is not apparent, some extra cracks may also develop in stage III.

The results of uniaxial tensile tests are presented in Table 4. An effective factor (EF) is used to highlight the bond properties. This is determined by dividing the peak load of CTRM specimens by that of corresponding carbon textile strips during the uniaxial test [25]. EF < 1 corresponds to a weak bond property, whereas EF > 1 indicates the existence of strain-hardening, namely, stage III in the stress–strain curve of the TRM specimens (Figure 6).

### 3.1. Influence of Reinforcement Ratio on the TRM Tensile Behavior

**Figure 7:**
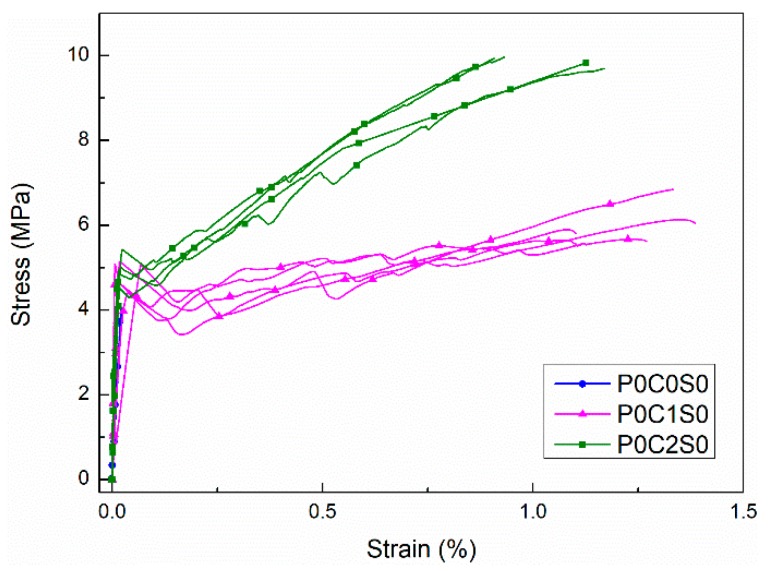
Stress–strain curves of the test specimens: P0C0S0, P0C1S0, and P0C2S0.

Figure 7 shows the stress–strain curves that were obtained from the tensile tests for specimens P0C0S0, P0C1S0, and P0C2S0. The stress–strain behavior of P0C0S0 is linear-elastic before reaching the ultimate stress. The stress then drops to zero after the brittle failure, indicating that only one crack forms in this specimen. Nevertheless, for P0C1S0 and P0C2S0, the stress increases approximately linearly with the strain until the first crack appears in the TRM specimens. An apparent drop of tensile stress occurs after reaching the first-crack stress in both P0C1S0 and P0C2S0 and then the stress continues to increase until dropping again upon the formation of a new crack. This procedure is repeated until the ultimate stress is reached, at which point the specimens fail completely.

**Figure 8:**
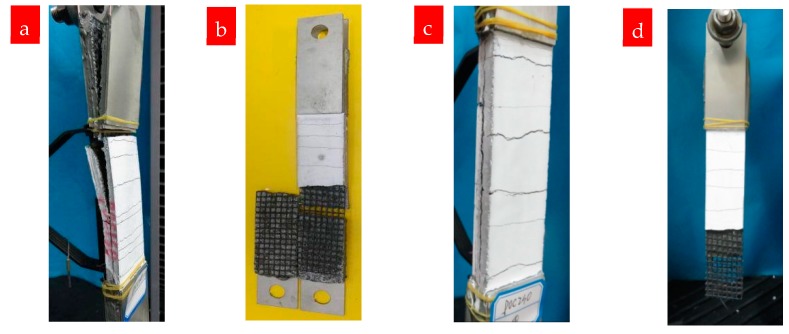
Debonding failure of TRM specimens: (**a**,**b**) P0C1S0 and (**c**,**d**) P0C2S0.

Generally, the tensile strength of both P0C1S0 and P0C2S0 increases with the reinforcement ratio. The average tensile strengths of P0C1S0 and P0C2S0 composites were 6.04 MPa and 9.88 MPa, respectively, representing increases of 0.47% and 1.41% over the unreinforced specimen (P0C0S0). The EF value of P0C1S0 was ~0.60, and that of P0C2S0 was ~0.49. Consistent with the specimen failure modes observed in Figure 8, the textiles did not break when the specimens failed, however became separated from the matrix, resulting in debonding failure and low utilization rate of carbon textiles. Figure 8 further reveals that the longitudinal debonding along the yarns parallel to the load direction occurred gradually as the load increased. Moreover, the tensile response of P0C1S0 and P0C2S0 exhibits a bilinear behavior (Figure 7). Stage III, typically observed in TRMs, did not occur in P0C1S0 and P0C2S0 because of the debonding failure mode. The lack of hardening in stage III is caused by the poor interfacial properties of TRM. Thus, practical measures should be taken to enhance the bond strength between the textiles and the matrix.

**Figure 9:**
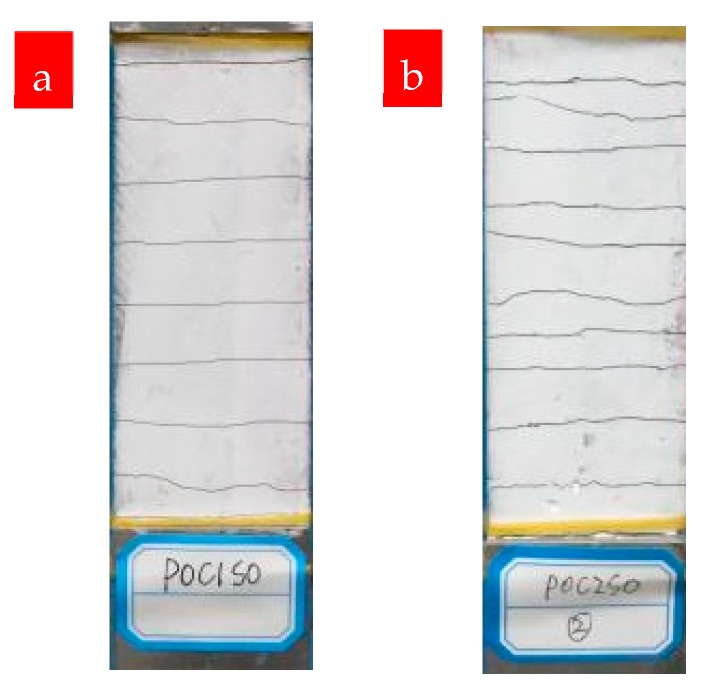
Cracking in TRM specimens: (**a**) P0C1S0 and (**b**) P0C2S0.

**Figure 10:**
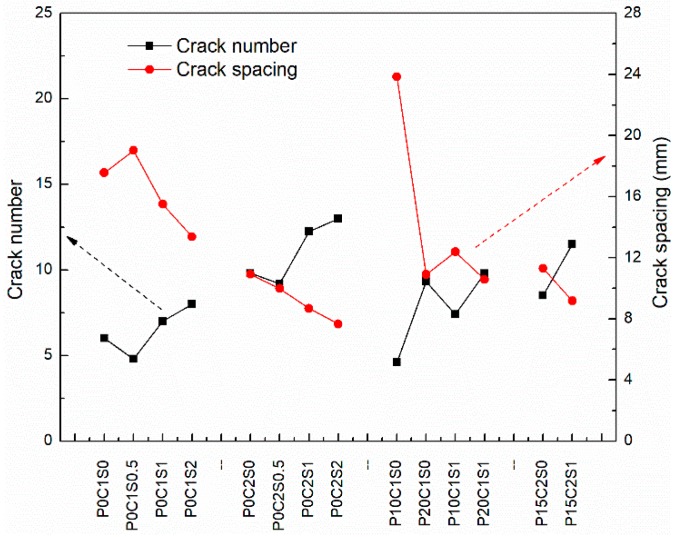
Crack number and spacing of TRM specimens.

The cracking patterns of P0C1S0 and P0C2S0 after uniaxial tensile tests are shown in Figure 9. Compared with the plain matrix, Figure 9 indicates that the TRM specimens possessed a uniform distribution of fine cracks. The increase in reinforcement ratios also affected the crack patterns. As shown in Figure 10 and Table 4, the number of cracks increased from 6 to 9.8 as the reinforcement ratio increased from 0.4% to 0.8%, accompanied by a reduction in the distances between cracks and the crack widths. Based on Figure 7 and Figure 8, the cracking mechanisms of P0C1S0 and P0C2S0 under tensile loading can be described as follows. (i) The first crack formed in the TRM composites when the tensile stress of the specimens reached the tensile strength of the cementitious matrix. (ii) The load originally carried by the matrix was transferred to the carbon textiles located in the crack. (iii) The bond strength between the matrix and textile allowed the textiles to transfer the load to the uncracked matrix located at either side of the crack. (iv) A new crack formed in the TRM when the stress in the uncracked matrix reached its tensile strength. (v) The stress constantly transferred between the textiles and the uncracked matrix and a multi-cracking pattern formed. (vi) No sequent cracks formed in the specimens when the interfacial bond property was so poor that the stress could not be transferred. (vii) Finally, the specimens suffered debonding failure.

### 3.2. Effect of Steel Fibers

**Figure 11:**
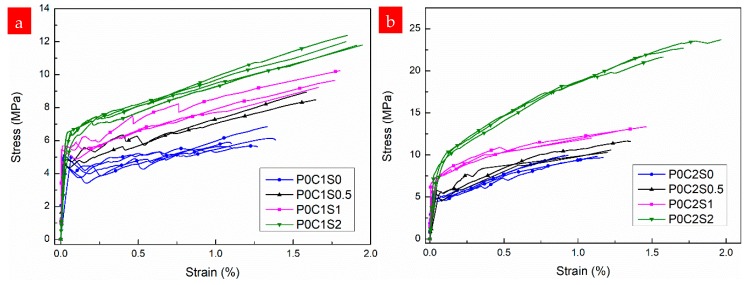
Stress–strain curves of the TRM specimens with varying volume fractions of short steel fibers: (**a**) one-layer and (**b**) two-layer textiles.

**Figure 12:**
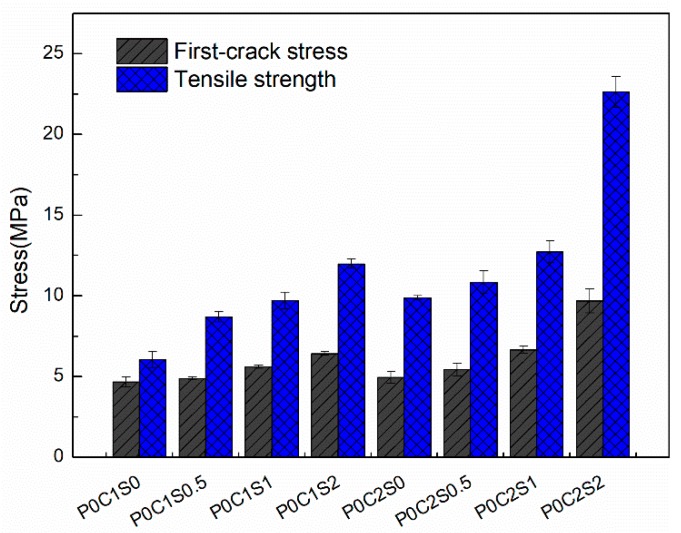
First-crack stress and tensile strength of the TRM specimens.

Figure 11 shows the experimental stress–strain responses of the TRM composites (with one or two layers of textile reinforcement) without and with short steel fibers in proportions of 0.5%, 1%, and 2% by volume. The first-crack stress and tensile strength of the TRM specimens without and with steel fibers are depicted in Figure 12. The numerical values, including the first-crack stress, uniaxial tensile strength, ultimate strain capacity, crack number, crack spacing, and EF values, are summarized in Table 4. Moreover, the stress–strain curves of the TRM with steel fibers are generally above those of the TRM without steel fibers, as shown in Figure 11. Therefore, the bearing capacity of TRM increases noticeably through the addition of steel fibers. Within the scope of this test, the improvements in tensile mechanical behavior were significantly correlated with the proportion of steel fibers.

From the experimental results, it can be inferred that short steel fibers distributed randomly in the grids of the textiles as secondary reinforcement improve the bond strength between the textiles and the matrix. The excellent bond strength is mainly attributable to the “shear resistant ability” of the steel fibers inserted vertically or obliquely into the grids of the textiles. Adding steel fibers to TRM could improve the cracking resistance of the matrix and further enhance the bearing capacity of the composites. These improved mechanical properties are characterized by a higher first-crack stress, smaller reduction in stiffness after cracking, smaller fluctuations in the stress–strain curves, and higher ultimate tensile strength compared with the specimens without steel fibers (Figure 11). 

With respect to the TRM reinforced with a single layer of textile and with 0.5%, 1%, and 2% steel fibers by volume, the ultimate tensile strength increased by 0.44%, 0.61%, and 0.98%, respectively, compared with the TRM specimens without steel fibers (Figure 12, Table 4). The strain capacity increased remarkably as a result of the strong bridging action of the steel fibers in cracks, and the maximum strain capacity of the TRM reinforced with one layer of textile was observed to increase by 1.89%.

The positive effects of steel fibers on the mechanical performance of TRM reinforced with two-layer textiles are clearly noticeable in Figure 11b. Significant improvements occurred in all mechanical properties of P0C2S2 compared with those of P0C2S0, with a 129% increase in ultimate tensile strength, 95.8% increase in first-crack stress, and 64.1% increase in strain capacity. However, P0C2S0.5 exhibited only a moderate increase in tensile strength. A slight increase in the first-crack stress of P0C2S0.5 compared with that of P0C2S1 and P0C2S2 was also observed. These findings can be attributed to the addition of steel fibers, enabling the textile to bond with the matrix. Clearly, adding higher proportions of steel fibers results in better bond properties. Improved mechanical properties are limited by the distribution and orientation of the short fibers. The number and extent of fluctuations in the curves decreased with the increasing proportion of steel fibers, indicating that the bond between the textile and the matrix was better for TRM specimens with a higher proportion of steel fibers. 

**Figure 13:**
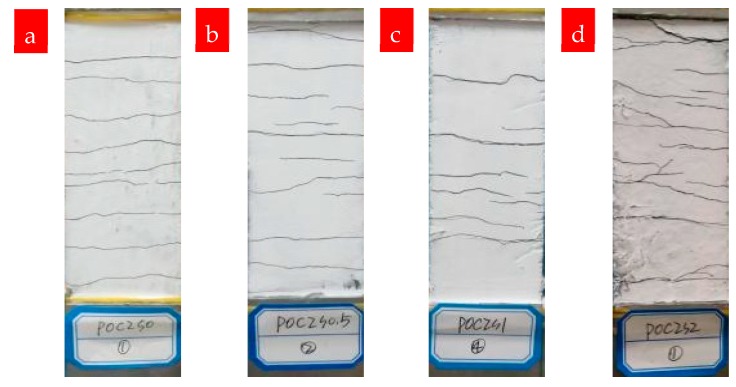
Cracking in TRM specimens with different volume fractions of steel fibers: (**a**) P0C2S0, (**b**) P0C2S0.5, (**c**) P0C2S1, and (**d**) P0C2S2.

Figure 13 compares the cracking patterns of P0C2S0, P0C2S0.5, P0C2S1, and P0C2S2, and clearly shows the differences resulting from varying steel fiber proportions. The visual surface inspection of the TRM specimens found a large number of micro-cracks in TRM specimens with steel fibers. The cracking patterns were also transformed from relatively straight and flat continuous cracks to irregular, short cracks. The cracks propagated along a more complex path, growing not only along the width of the specimen, however also along the length. The steel fibers, distributed randomly in the grids of the textiles, enabled some resistance to micro- and macro-crack propagation and changed the direction of the development of the cracks as well as the cracking patterns. 

**Figure 14:**
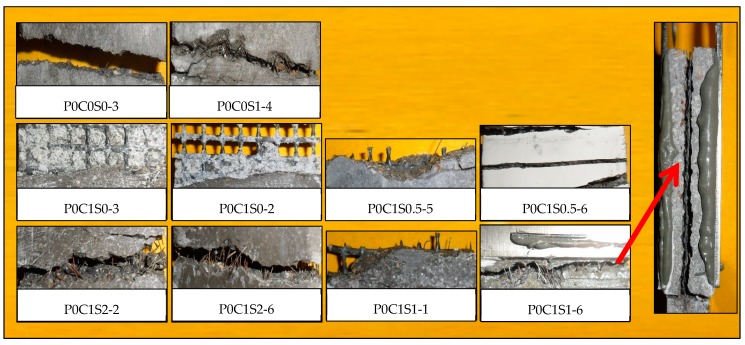
Fracture behavior of plain matrix, matrix reinforced with 1% steel fibers, and TRM (one-layer) specimens with steel fibers.

**Figure 15:**
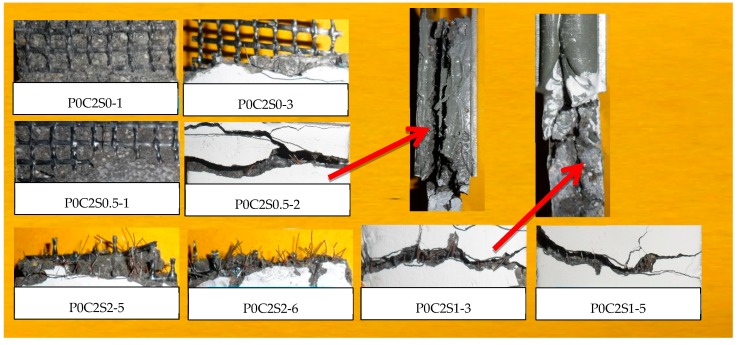
Fracture morphology of TRM specimens with steel fibers and two-layer textiles.

The fracture surfaces of TRM specimens with steel fibers are shown in Figure 14 and Figure 15. For both P0C1S1and P0C2S1, only a small number of specimens failed because of the complete fracture of carbon textiles. Most of the specimens still exhibited debonding failure. With the increase in the steel fiber proportions in the TRM, the failure mode of both P0C1S2 and P0C2S2 transformed into a complete fracture of carbon textiles, i.e., a high utilization rate of carbon textiles was achieved. Adding 2% steel fibers to the TRM composites can be regarded as an effective means of enhancing the textile–matrix bond property. Additionally, it can be seen in Figure 10 that P0C2S2 had the maximum crack number as well as the minimum crack spacing among all the specimens. P0C2S2 had an average of 13 cracks and an average crack spacing of 7.66 mm. 

**Figure 16:**
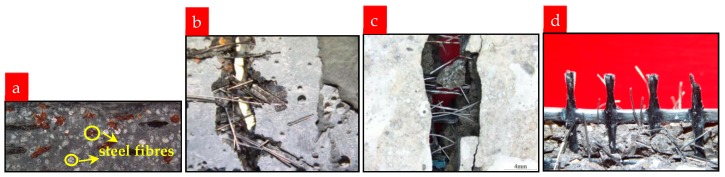
(**a**) Well-distributed steel fibers; bridging capacity of steel fibers in cracks: (**b**) P0C1S1, (**c**) P0C2S1; (**d**) fracture surface of TRM with steel fibers.

**Figure 17:**
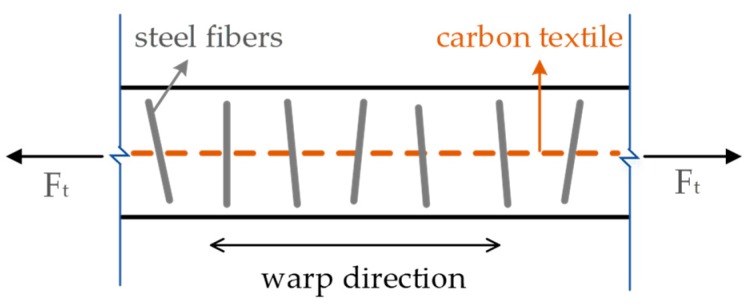
Side view of steel fiber distribution in the CTRM (carbon textile reinforced mortar).

The improvements in strength and failure behavior of TRM resulting from the addition of steel fibers may be ascribed to the following mechanisms:

1. As shown in Figure 16a, the steel fibers are evenly distributed throughout the cross-section of the specimen. Well-distributed steel fibers form a good bond with their surrounding matrix and further reduce the shrinkage of the matrix, thus reducing the resulting internal defects in the matrix. Moreover, steel fibers also inhibit the formation of micro-cracks, which develop because of shrinkage.
2. Steel fibers play a bridge role in micro-cracks and delay the formation of the first macro-crack. Thus, higher stress is needed to cause the transition from micro-cracks to macro-crack. The bridging of steel fibers in micro-cracks contributes to the improved first-crack stress of TRM specimens with steel fibers. In addition, the bridging action of steel fibers in macro-cracks causes additional stress transfer over the cracks (Figure 16b,c); thus, a new crack can be formed at a smaller distance from an existing crack. Thus, fine multiple cracking can develop and lead to pronounced ductility.
3. The elastic modulus of carbon textile is greater than that of mortar matrix, so their deformation after tensioning is different, causing a relative sliding trend to occur. For TRM composites without the addition of short fibers, the bond properties between the textile and the cementitious matrix depend on the friction and adhesive caused by the matrix hydration products. For TRM composites with the addition of steel fibers, the bonding properties are enhanced. Steel fibers inserted into the textile grids can mitigate the trend, enabling a better cooperation of the carbon textile and mortar. As shown in Figure 17, steel fibers there can resist shearing force. Hence, it can be summarized that steel fibers in the textile grids provide additional "shear resistant ability" between the carbon fabric and the mortar, thus improving the bonding properties between carbon fabric and inorganic mortar in CTRM. Investigation of the fracture surfaces of TRM with steel fibers using an optical microscope has helped to explain this new link (Figure 16d).

### 3.3. Effect of Prestress

**Figure 18:**
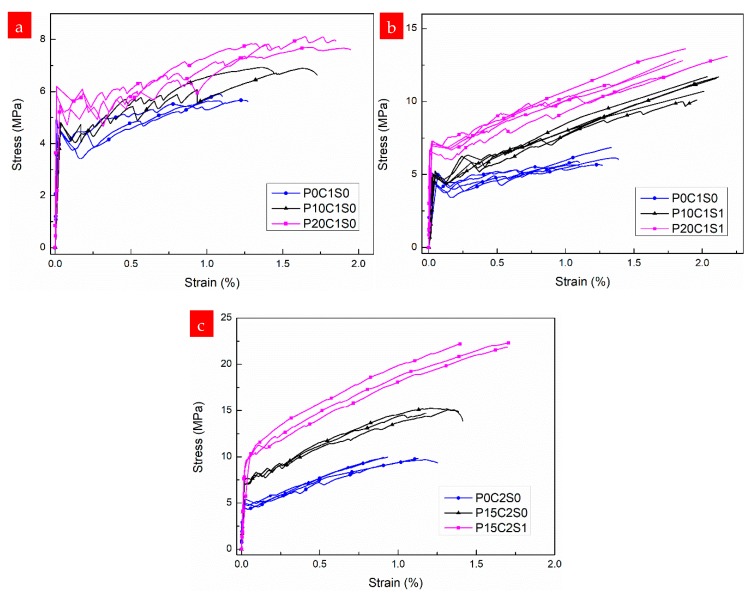
Stress–strain curves of the prestressed TRM specimens: (**a**) reinforced with one-layer textile and without steel fibers, (**b**) reinforced with one-layer textile and 1.0% steel fibers, and (**c**) reinforced with two-layer textiles.

**Figure 19:**
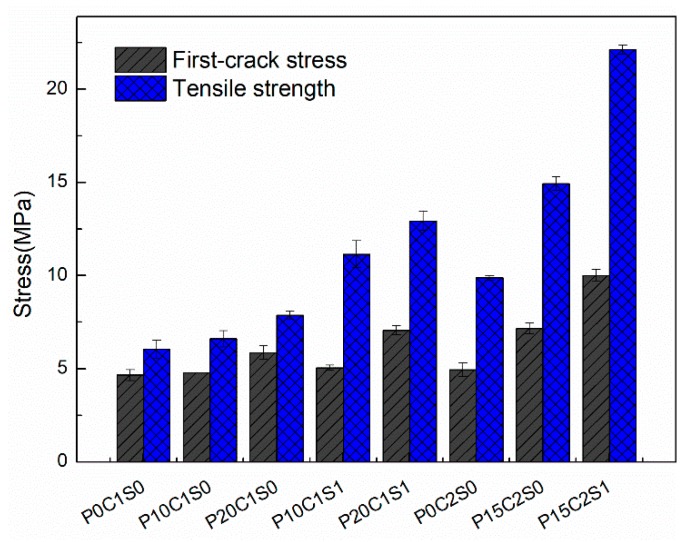
First-crack stress and tensile strength of the prestressed TRM specimens.

Figure 18 presents the stress–strain curves of the prestressed TRM specimens. The results, including a summary of the tensile strength, ultimate strain, first-crack stress, crack number, crack spacing, and EF values are listed in Table 4. The first-crack stress and tensile strength of the prestressed TRM specimens are depicted in Figure 19. According to the results shown in Figure 18a, pre-tension to 10% and 20% of the ultimate tensile capacity of the one-layer textile increased the specimens’ first-crack stress. Although both the first-crack stress and tensile strength improved with increases in the prestress level, the improvements are not obvious (Figure 19). For example, the first-crack stress and ultimate tensile strength of P20C1S0 only increased by 25.7% and 30.5%, respectively, compared with those in P0C1S0. Figure 18b shows that adding 1% steel fibers to the prestressed TRM specimens improves their mechanical properties. The first-crack stress, ultimate tensile strength, and strain capacity of P20C1S1 increased by 51.5%, 114%, and 57.4%, respectively, compared with those in the reference specimen P0C1S0. 

According to the results presented in Figure 18c and Figure 19, TRM specimens pre-tensioned to 15% of the ultimate tensile capacity of the two-layer textiles (P15C2S0, P15C2S1) exhibit higher first-crack stress and tensile strength than the control TRM specimen (P0C2S0). Compared with P0C2S0, the first-crack stress of P15C2S0 and P15C2S1 increased by 44.8% and 102%, and the tensile strength increased by 51.2% and 124%, respectively. The average crack number and crack spacing of P15C2S1 increased from 9.8 and 11.92 mm to 11.5 and 9.18 mm in comparison with P0C2S0 (Figure 10 and Table 4). It can be concluded that adding 1% steel fibers by volume to prestressed TRM specimens (P15C2S1) is an effective method of improving the specimens’ mechanical performance. 

**Figure 20:**
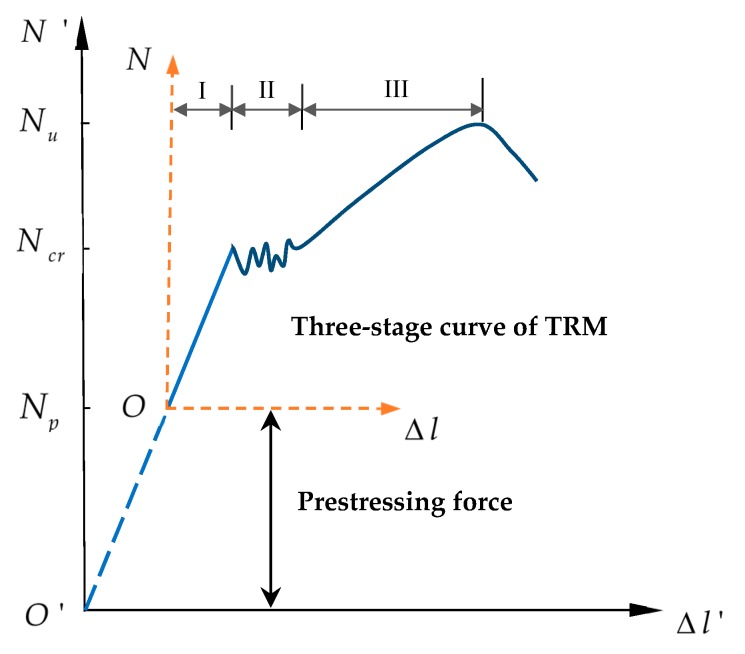
Influence of prestress on TRM.

The effect of prestress can be explained by referring to Figure 20. Point O is the origin of the tension force N with respect to displacement Δl. The curve includes three distinctive stages, i.e., elastic, multiple cracking, and post-cracking. In this case, $N_{cr}$ indicates the critical tensile load at which the mortar matrix first cracks, corresponding to the moment that the tensile load is mostly transferred to the textiles, whereas $N_{u}$ is the ultimate tensile load, beyond which the specimen loses its load capacity. Whether the slope of the post-cracking stage is steeper or shallower than that in stage I depends on the stiffness of the reinforcing textiles. Once the textiles in the TRM specimen are pre-tensioned, the origin O shifts to point $O^{\prime }$, permitting the previous short uncracked stage to extend to an ideal duration [26]. With respect to this particular curve, a prestressing force is exerted on the textiles at point $O^{\prime }$ and released at point $N_{p}$. Thus, an initial compressive stress on the concrete matrix was achieved, leading to an increase in the first-crack stress [35]. Moreover, after releasing the prestress on the mortar matrix, the bond strength between the textile and matrix is considerably improved, so the ultimate tensile strength of the TRM specimens also increases (Figure 18 and Figure 19). The development process after releasing the prestress in TRM specimens runs along the same path as the un-prestressed specimens until the final failure. As a result, exerting a prestressing force on the textiles extends the serviceability limit states of TRM and produces more reliable workability. 

**Figure 21:**
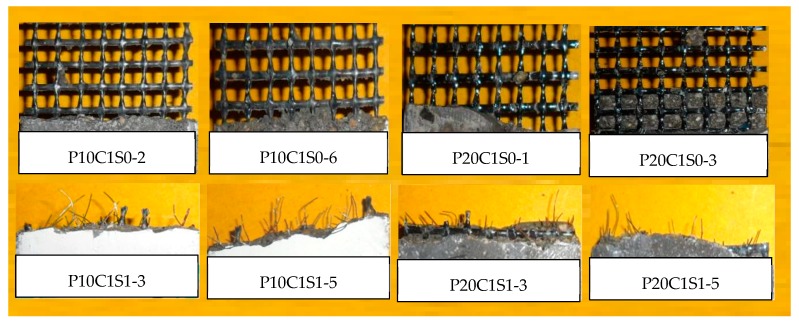
Fracture morphology of prestressed TRM specimens with one-layer textile.

**Figure 22:**
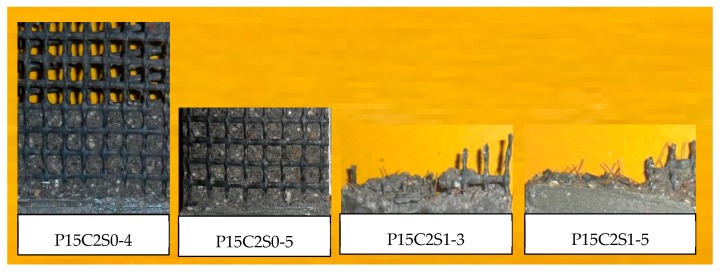
Fracture morphology of prestressed TRM specimens with two-layer textiles.

Although prestressing the textiles improves the cooperative bearing ability between the textile and the matrix to a certain extent, specimens P10C1S0, P20C1S0, and P15C2S0 exhibited debonding failure. The failure modes of P10C1S1, P20C1S1, and P15C2S1 changed from debonding to the complete fracture of carbon textiles; thus, adding 1% steel fibers by volume to prestressed TRM specimens could significantly improve the textile–matrix bond properties. The fracture morphologies of prestressed TRM specimens are shown in Figure 21 and Figure 22. 

## 4. Conclusions

In this research, the effects of the reinforcement ratio, short steel fibers as additional reinforcements, and prestressing have been explored with regard to the strength and failure behavior of TRM subjected to tensile loading. The following conclusions can be drawn:

(1) Generally, the tensile strength of both P0C1S0 and P0C2S0 increased with an increase in the reinforcement ratio. However, the EF values of specimens P0C1S0 and P0C2S0 decreased as the reinforcement ratio increased, indicating weakened textile–matrix bond strength. The textiles did not snap when P0C1S0 and P0C2S0 failed, however slid from the matrix, thereby resulting in debonding failure and low utilization rate of the carbon textiles. In contrast, the utilization rate of carbon textiles increased remarkably when short dispersed steel fibers of 2% volume fraction were inserted into the grids of the textile.

(2) Short steel fibers are able to improve the mechanical properties of the mortar and the entire composite in experiments. Moreover, steel fibers improve the textile–matrix bond strength, which can be attributed to the “shear resistant ability” of steel fibers inserted into the grids of the textile. Increases in tensile strength were clearly observed in all specimens with added steel fibers. An investigation of the fracture surfaces using an optical microscope further revealed that short steel fibers added to TRM cause finer cracks with smaller spacings and widths. Furthermore, within the scope of this test, the improvements in tensile mechanical behavior were highly correlated with the increase in the steel fiber proportion. Compared with the results that were obtained for the reference TRM plates, the tensile strength increased by approximately 100% following the addition of 2% steel fibers by volume. 

(3) Increases in first-crack stress and tensile strength were also observed in prestressed TRM specimens. The enhanced first-crack stress was attributed to the extension (caused by pre-compression of the mortar matrix after being released) of stage I, i.e., the uncracked state corresponding to the related mortar matrix. The tensile strength increased as the bond behavior improved, a result of the strengthened interaction effect between the surface of the textile and the matrix activated by prestressing. Therefore, the serviceability limit states of TRM composites can be extended by exerting a prestressing force on the textiles.

(4) Adding steel fibers at 1% volume to prestressed TRM specimens is an effective method of improving the specimens’ mechanical performance, dramatically enhancing the bond strength between matrix and textiles. As a result, the failure mode changes from debonding to the complete fracture of the carbon textiles. In this study, the combination of 1% steel fibers and prestress calculated at 15% of the ultimate tensile strength of the two-layer textiles was found to be the optimum configuration, producing the highest first-crack stress and tensile strength and the most reasonable multi-cracking pattern.

## Figures and Tables

**Table 1:** Physical and mechanical properties of the warp carbon yarns (impregnated).

Type	Tensile Strength (MPa)	Young’s Modulus (GPa)	Strain Capacity (%)	Density (g/cm^3^)	Cross-Sectional Area (mm^2^)	Tex (g/km)
6K	2290	230	1	1.8	0.218	390

Note: 6K means that one multifilament yarn consists of 6000 monofilaments.

**Table 2:** Mechanical properties and geometric parameters of steel fibers.

Diameter (mm)	Length (mm)	Density (g/cm^3^)	Tensile Strength (MPa)	Young’s Modulus (GPa)
0.18–0.23	12–15	8.5	2850	200

**Table 3:** Composition of TRM (textile reinforced mortar) matrix.

Materials	Cement Type II 52.5	Fly ash	Silica Fume	Slag	Fine Sand	Super-Plasticizer	Water
Contents (kg/m^3^)	800	100	50	50	1200	2.0	286

**Table 4:** Mechanical properties of uniaxial tensile tests.

Specimen	First-crack Stress (MPa)	Tensile Strength (MPa)	Strain Capacity (%)	Crack Number (/)	Crack Spacing (mm)	EF (/)
P0C1S0	4.66 (0.30)	6.04 (0.49)	1.22 (0.13)	6 (2.10)	17.56 (1.86)	0.60 (0.20)
P0C1S0.5	4.88 (0.10)	8.71 (0.32)	1.61 (0.04)	4.8 (0.84)	19.03 (2.67)	0.87 (0.13)
P0C1S1	5.61 (0.11)	9.71 (0.51)	1.74 (0.07)	7 (1.58)	15.51 (2.55)	0.97 (0.20)
P0C1S2	6.43 (0.11)	11.99 (0.28)	1.89 (0.05)	8 (1.58)	13.37 (3.03)	1.20 (0.11)
P0C2S0	4.95 (0.36)	9.88 (0.12)	1.03 (0.13)	9.8 (2.39)	10.92 (3.46)	0.49 (0.05)
P0C2S0.5	5.43 (0.40)	10.84 (0.71)	1.25 (0.07)	9.2 (2.59)	9.98 (2.25)	0.54 (0.28)
P0C2S1	6.65 (0.23)	12.72 (0.68)	1.27 (0.18)	12.25 (2.36)	8.68 (2.49)	0.64 (0.27)
P0C2S2	9.69 (0.75)	22.63 (0.96)	1.69 (0.11)	13 (2.64)	7.66 (2.12)	1.13 (0.38)
P10C1S0	4.80 (0.002)	6.61 (0.44)	1.45 (0.10)	4.6 (1.14)	23.84 (2.89)	0.66 (0.18)
P20C1S0	5.86 (0.36)	7.88 (0.20)	1.58 (0.25)	9.33 (2.08)	10.9 (3.41)	0.79 (0.08)
P10C1S1	5.06 (0.13)	11.15 (0.72)	2.04 (0.07)	7.4 (1.52)	12.39 (1.79)	1.12 (0.29)
P20C1S1	7.06 (0.25)	12.92 (0.51)	1.92 (0.15)	9.8 (1.92)	10.57 (2.56)	1.29 (0.20)
P15C2S0	7.17 (0.30)	14.94 (0.37)	1.18 (0.14)	8.5 (1.29)	11.31 (2.39)	0.75 (0.15)
P15C2S1	10.01 (0.32)	22.12 (0.25)	1.60 (0.18)	11.5 (3.70)	9.18 (2.320)	1.11 (0.10)

Note: Values in parentheses denote the standard deviations.
